# Comparison of Antibacterial and Antioxidant Properties of Red (cv. Negramaro) and White (cv. Fiano) Skin Pomace Extracts

**DOI:** 10.3390/molecules26195918

**Published:** 2021-09-29

**Authors:** Carmela Gerardi, Loris Pinto, Federico Baruzzi, Giovanna Giovinazzo

**Affiliations:** 1 Institute of Sciences of Food Production, National Research Council of Italy, via Lecce-Monteroni, 73100 Lecce, Italy; carmela.gerardi@ispa.cnr.it; 2 Institute of Sciences of Food Production, National Research Council of Italy, via G. Amendola 122/O, 70126 Bari, Italy; loris.pinto@ispa.cnr.it (L.P.); federico.baruzzi@ispa.cnr.it (F.B.)

**Keywords:** antimicrobial, antioxidant, phenol, grape by-products, food pathogens

## Abstract

Wine pomace has attracted the attention of the food industry, due to its high content in bioactive compounds, and its multiple healthy activities. In this work, whole and separated skin pomaces from fermented (red) and un-fermented (white) grape by-products were characterized for their antioxidant and antimicrobial activities in order to exploit them as functional food ingredient. Antioxidant activity, measured by both ORAC and TEAC assays, was higher in whole than in skin pomace extracts. The characterization of phenolic composition in whole and skin pomace extracts confirmed the peculiarity of some compounds such as anthocyanins (107.84 + 10.3 mg/g TP) in red skin pomace and a great amount of flavanols (80.73 + 4.04 mg/g TP) in white skin pomace. Whole and skin pomace extracts displayed the same antibacterial activity at 250 µg gallic acid equivalents (GAE)/mL. Red and white skin pomace extracts showed a Minimum Inhibitory Concentration (MIC) of 31.25–62.5 GAE/mL against *Staphylococcus aureus* and *Enterococcus faecalis*. *Pseudomonas* spp. were more sensitive to red skin pomace extracts rather than white skin pomace extracts. Given these results, both red and white pomace extracts could be exploited for future application in food, pharmaceutical and cosmetic industry.

## 1. Introduction

The health-promoting activities of plant polyphenols have been widely ascertained by hundreds scientific publications. Studies on plant extracts and phytochemicals showed that polyphenols could play an anti-inflammatory and antibacterial action [1,2,3,4]. Grape by-products are one of the main sources of bioactive polyphenols [5,6]. In particular, grape pomace polyphenols are endowed of antiallergenic, anti-inflammatory, anticancer, anti-ageing, antimicrobial, antioxidant, antithrombotic, insulinotropic, antilipotropic, cardioprotective and vasodilatory activity [7].

However, the bioactivities of grape pomace extracts are the result of their polyphenolic profile that is affected by the grape variety, the geographical origin and the winemaking process [1,8,9]. The phenolic compounds of grape pomace can be classified in phenolic acids, flavonoids (flavonols, flavanols, anthocyanins), stilbenes, and tannins [5]. The dried grape pomace or purified extract of grape pomace could be recommended to overcome potential limitations of grape pomace utilization, such as toxicity, storage stability, and the sustainability of the recovery method. Several innovative extraction methods for the recovery of polyphenols with antioxidant, antimicrobial and other biological properties from different grape pomace extracts have been recently explored [7,10,11]. As far as their antioxidant activity is concerned, Gerardi et al. [10] found that ultrasound- and microwave-assisted extraction of pomace skin (Negramaro and Primitivo cultivars) using acidified water allowed the highest Trolox equivalents antioxidant capacity values. Flavan-3-ols, phenolic acids and ethyl gallate strongly correlated with the scavenging activity of whole grape pomace extracted in acidified methanol [12]. In addition, Feteasca Neagra and Pinot noir grape pomace ethanolic extracts significantly reduced the in vitro inflammation-induced oxidative stress in a concentration dependent way as well as the proliferation of four malignant cell lines [13].

A number of studies have been carried out on the antibacterial effect of grape pomace extracts against foodborne pathogens. The antimicrobial effect of grape pomace extracts is usually ascribed to their complex phenolic composition. Several studies have shown that the main phenolic compounds involved in the antibacterial activity are the phenolic acids rather than the flavonoids. In particular, high potential of hydroxycinnamic acids rather than hydroxybenzoic acids to penetrate the cell membrane was reported [14]. Recently, total flavan-3-ols strongly correlated with the antibacterial activity displayed by grape pomace extracts [15]. In addition, polyphenols of red grape pomace extract potentiated the effects of various classes of antibiotics against *Staphylococcus aureus* and *Escherichia coli*, resulting particularly useful to control the growth of multi-drug resistant clinical isolates [16,17].

The antibacterial activity of grape pomace extracts against spoilage microbial populations, as for other wine by-products, mitigated the negative effects of microbial metabolisms, such as gas formation [18,19], slime formation [20], acid production [21] and the production of biogenic amines [22,23]. Therefore, grape pomace can be exploited as natural preservative in the food sector for improving food quality and safety.

Despite these results, the comparison of antioxidant and antimicrobial action of grape pomace extracts from different cultivars and the correlation with their phenolic profile needs further investigation. In this work, grape pomace extracts were obtained from whole pomaces or berry skins belonging to red (Negramaro) and white (Fiano) grape cultivars. The antioxidant and antibacterial activity of lyophilized hydro-alcoholic extracts was evaluated. The phenolic profile of these extracts was determined by means of chromatographic analyses.

## 2. Results

### 2.1. Antioxidant Activity and Phenolic Profiles of Whole and Skin Pomace Extracts

Total phenol content in whole pomace extracts was higher than that found in skin pomace extracts (Table 1). Antioxidant activity of whole and skin pomace extracts showed different values employing TEAC or ORAC assays. In particular, pomace extracts from Negramaro cultivar showed ORAC values higher than TEAC values (Table 1). Antioxidant activity of WP extracts showed higher TEAC and ORAC values than SP extracts for both cultivars. However, WPN and WPF samples showed comparable values, whereas SPN and SPF samples were significantly different for the total antioxidant activity (Table 1). Indeed, Negramaro skin pomace showed higher values for antioxidant activity (651.315 ± 26.91 μmol TE/g d.w. TEAC and 1337.75 ± 149.4 μmol TE/g d.w. ORAC) than white skin pomace. The skin pomace samples have a similar total phenol content, but differ substantially in the amount of the individual compound or classes of phenols as shown in Table 2.

The analysis of phenols in whole pomace samples (WPN and WPF in Table 2) showed that flavonols in both Negramaro and Fiano whole pomace were lower than corresponding skin samples. The characterization of phenols in skin extracts showed high concentration of specific molecules such as anthocyanins in SP extract of Negramaro red pomace and flavanols in SP extract of Fiano white pomace (SPN and SPF in Table 2). This finding could be explained by the dilution effect in the whole pomace extract since the synthesis of many compounds resides in the grape skin. Taking into account that the peel represents 5% (*w/w*) of the dried whole pomace, some compounds are very concentrated in the peel extracts and in some cases not detected in the whole samples.

The highest values of ORAC were found in both whole and skin red pomace (Table 1). This result is probably due to the synergy of many active molecules representing different families such as flavanols, flavonols and anthocyanins (Table 2).

With TEAC higher values of antioxidant activity were found in samples of whole pomace where flavanols are abundant. The SPN sample had significantly higher TEAC and ORAC values than SPF (Table 1). This result could be ascribed to the presence of anthocyanins and to the synergy between the different groups of molecules.

### 2.2. Antibacterial Activity of Pomace Extracts

Whole and skin pomace extracts were used at the same concentration of total polyphenol content in antibacterial assays. As regards whole and skin pomace extracts of the cultivars Fiano and Negramaro, these samples (250 µg GAE/mL) inhibited the growth of *S. aureus* DSM 799 and *E. faecalis* ATCC 47077, showing a different antibacterial activity against *Pseudomonas* spp. strains. Indeed, whole and skin pomace extracts of the cultivar Fiano (250 µg GAE/mL) delayed the growth of all strains, wheras extracts of the cultivar Negramaro, at the same concentration, inhibited the growth of *Pseudomonas fluorescens* NCPPB 1964^T^ and *P. putida* ITEM 17297, and delayed the growth of *P. chicorii* ITEM 17296 and *P. aeruginosa* DSM 939 (data not shown). Overall, whole and skin pomace extracts of both cultivars displayed the same antibacterial action at 250 µg GAE/mL. Skin pomace extracts showed great amount of phenolic acids and flavonols, in some cases higher than whole pomace extracts. Moreover, skin pomace extracts showed higher concentration of total flavonoids than whole pomace extracts (Table 2). Given these results, MIC values and growth parameters were determined only for skin pomace extracts against all bacterial strains.

The SP extracts showed different antibacterial activity against Gram-positive and Gram-negative bacteria. The SPF extract showed a MIC of 62.5 µg/mL and 31.25 µg/mL total polyphenols against *S. aureus* DSM 799 and *E. faecalis* ATCC 47077, respectively. At the MIC level and at the SPF concentration of 250 µg GAE/mL, a reduction of the cell viability higher than 5 orders of magnitude was found for these bacteria.

The SPN showed a MIC of 62.5 µg GAE/mL against *S. aureus* DSM 799 and *E. faecalis* ATCC 47077. However, at the end of incubation, a reduction of cell viability higher than 5 log cfu/mL was found only for *S. aureus* DSM 799. Viable cell counts of *E. faecalis* ATCC 47077 showed mean values of 11.70 ± 0.05 log cfu/mL in mPCB and of 9.40 ± 0.05 log cfu/mL in mPCB amended with SPN.

*Pseudomonas* spp. were partially inhibited by SPF at 250 µg GAE/mL. As reported in Table 3A, the SPF extract at this concentration delayed the growth of *Pseudomonas* strains, reducing the maximum growth rate and the maximum optical density, both calculated during 24 h of incubation. At the same time, the doubling time increased. No relevant changes were detected in the lag time values (Table 3A). At the end of incubation, viable cell counts of *Pseudomonas* spp. treated with the SPF250 µg GAE/mL did not show differences in comparison to control samples.

As example the growth kinetic affected by SP exposure of *Pseudomonas* spp. is shown in Figure S1, Supplementary Materials.

*Pseudomonas* spp. were more sensitive to SPN than SPF. Indeed, SPN showed a MIC of 250 µg GAE/mL against *P. fluorescens* NCPPB 1964^T^ and *P. putida* ITEM 17297. In the case of *P. putida*, this concentration corresponded to the Minimum Bactericidal Concentration (MBC). At the end of incubation, a significant reduction of cell viability was found only for *P. fluorescens* NCPPB 1964^T^. Due to the antibacterial activity produced by SPN against these strains at 250 µg GAE/mL, the change in growth kinetic parameteres for these strains was measured at 125 µg GAE/mL whereas the concentration of 250 µg GAE/mL was employed for *P. chicorii* ITEM 17296 and *P. aeruginosa* DSM 939. At these concentrations, SPN significantly reduced the maximum growth rate and maximum optical density, whereas it significantly increased the lag time and the doubling time (Table 3B). After 24 h at 30 °C, SPN at 250 µg GAE/mL strongly reduced the viable cell count of *P. fluorescens* NCPPB 1964^T^. Indeed, a mean value of 11.75 ± 0.01 log cfu/mL in mPCB and of 3.00 ± 0.05 log cfu/mL in mPCB amended with SPN was found. On the contrary, the treatment with SPN at 250 µg GAE/mL did not reduce the viable cell counts of *P. chicorii* ITEM 17296 and *P. aeruginosa* DSM 939.

## 3. Discussion

In this work, the antioxidant and antibacterial activity of pomace extracts from red and white grape cultivars has been compared, with a focus on their phenolic profile. Total phenol content (TP) of whole and skin pomace extracts was not affected by the grape cultivar, showing comparable values in Negramaro cv (red) and Fiano cv (white) extracts (Table 1).

The comparison of antioxidant activity among whole and skin pomace extracts showed higher ORAC values for the red pomace of Negramaro cv (2496.75± 449.2 and 1337.75 ± 149.4 μmol TE/g d.w., for WPN and SPN respectively) than those detected for the white pomace of Fiano cv.

The value of total phenols was similar in the two cultivars but higher values were detected in whole pomace extracts than skin pomace extracts. However, SPN samples showed high antioxidant capacity as compared with SPF samples. This result is probably due to the presence of anthocyanins in the SPN sample. In some cases, it was found a strong correlation between the content of phenolic compounds in the final extract and its antioxidant activity. Other authors have verified that there is a strong correlation between total phenol content and antioxidant activity [24]. Although total phenolic content of grape pomace extracts correlated with their antioxidant activity, some phenolic compounds appear to confer a greater contribution to the antioxidant capacity of the extracts. While anthocyanins appear to make the main contribution to the antioxidant activity of SPN sample, the phenolic acids, flavanols and flavonols, seem to contribute significantly to the antioxidant capacity of whole pomace extracts, and SPF sample. Thus, after the determination of the total phenolic concentration, the comparison of some specific antioxidant activities related to polyphenolic compounds need to be carried out, starting from the the phenolic composition of the extracts [25]. Ky and Tessedre [24] suggested that the antioxidant activity of Mediterranean grape skin pomace extracts depends on the extraction (in water or in ethanol solution), the antioxidant assay employed (ORAC, FRAP, TEAC, DPPH), as well as the different grape variety. Xu et al. [26] demonstrated that different free radical scavengers positively correlated with different phenolic fractions of grape pomace extracts. Given these findings, it is difficult to correlate the total antioxidant capacity of grape pomace extracts to single polyphenolic fractions. The results confirmed our previous data showing that the aqueous extracts of white skin pomace contained the highest amount of flavonols [10]. Some classes of compounds such as soluble phenolic acids and flavonoids (flavanols, flavonols and anthocyanins) have been characterized in WPN, WPF, SPN and SPF samples, by means of RP-HPLC, using the total phenol content as a reference (Table 2). In particular, anthocyanins were the most representative polyphenols in the red skin extract, followed by phenolic acids, flavonols, and flavanols (Table 2). Conversely, in skin white grape pomace extract the flavonols resulted to be polyphenol fraction with the highest content, followed by flavanols and phenolic acids (Table 2). It can be assumed that skin pomace samples with a higher anthocyanin content have a higher antioxidant activity, as revealed by TEAC value of SPN extract.

The antibacterial activity of whole and skin pomace extracts from red and white cultivars depends on the total phenol concentration, the different phenolic profile, and the different resistance of the tested bacteria. Whole and skin pomace extract showed comparable antibacterial activity at 250 µg GAE/mL. On the basis of the specific phenolic composition of skin pomace extracts, their antibacterial action was further explored with the determination of MIC values and measurement of some microbial growth parameters. Our findings suggest that, the antimicrobial action of SPN against *Pseudomonas* spp. was higher than that acted by SPF (Table 3). MIC values of both samples against pathogenic and spoilage bacteria were in accordance with those found by Katalinić et al. [27]. These authors compared the antibacterial activity of SP from white and red cultivars, showing lower MIC values of extracts from white cultivars against Gram-negative bacteria than Gram-positive bacteria. Our data suggest higher antibacterial activity of SP against Gram-positive bacteria (*S. aureus* and *E. faecalis*) than Gram-negative bacteria (*Pseudomonas* spp.), as previously reported [28,29]. Red grape pomace extract and powder showed antibacterial activity against *Escherichia coli*, *S. aureus* and *Listeria innocua* [30,31]. Gram-positive bacteria were more sensitive to grape pomace treatment than Gram-negative bacteria [31,32]. These differences could be explained by the presence of the lipopolysaccharide cell wall in Gram-negative bacteria, which can limit the penetration of polyphenols into the cell.

The antimicrobial effect of grape pomace polyphenols has been associated to different mechanisms of action. Phenols can determine the cell membrane disruption and structural changes [32], as revealed in cell structures of *S. aureus* treated with grape seed extract [33]. The penetration of polyphenols into the cell of Gram-negative bacteria is hindered by the outer hydrophilic membrane. Grape pomace polyphenols may inactivate intracellular and extracellular enzymes [21] or damage microbial DNA [34]. Other potential mechanisms of action are the metal sequestration [35] and the formation of complexes with proteins, both affecting the membrane transport.

Differences in the antimicrobial activity of different extracts could be ascribed to their different phenolic profile, which is affected by the grape variety [36,37] and the extraction methods [38,39,40]. The major differences between the two extracts is the presence of anthocyanins and higher content of phenolic acids in the red grape extract (SPN) than SPF. These differences could determine a different antimicrobial action, in particular against Gram-negative bacteria such as *Pseudomonas* spp. Future works will be addressed to the identification of the active phenolic compounds and of possible synergistic activity among main and minor compounds occurring in these extracts.

In fact, individual phenolic compounds showed lower antibacterial activity than that exerted by grape pomace extracts or their powder, which suggest possible synergistic effects among different classes of phenolic compounds [31,41].

The results here reported open to the possibility to isolate and to deeply characterize antibacterial and antioxidant activity of specific fractions from red and white SP in order to exploit this wine making by-product for different applications in the food, pharmaceutical and cosmetic sectors.

## 4. Materials and Methods

### 4.1. Reagents and Standards

Reagents were acquired from various suppliers: authentic standards of oenin (Malvidin-3-*O*-glucoside), rutin (quercetin 3-*O*-rutinoside), chlorogenic acid (5-caffeoylquinic acid) from Extrasynthèse (Genay, France); gallic acid, caffeic acid, caftaric acid, coutaric acid, catechin, epicatechin, quercetin-3-glucoside, Folin-Ciocalteu phenol reagent, Trolox [(*S*)-(−)-6-hydroxy-2,5,7,8-tetramethylchroman-2-carboxylic acid], acetonitrile, formic acid, ethanol, (all HPLC grade) from Sigma-Aldrich (St. Louis, MO, USA). Milli-Q water (Merck Millipore, Darmstadt, Germany) was used for the preparation of reagents and antioxidant assays.

### 4.2. Raw Material and Sample Preparation

Two batches of wine pomace (both obtained in the late summer of 2019), *Vitis vinifera* L. varieties Negramaro (N), (achieved after fermentation for red wine making), and Fiano (F) (without fermentation, as it is used in white wine making) were obtained from a commercial winemaking facilitylocated in Salento (Cantine Cantele, Apulia Region, Southern Italy). Pomace samples were dried in an oven at 50 °C, until constant weight. Grape skins, manually separated from whole pomaces, were stored at −20 °C until further assays.

### 4.3. Preparation of Liophilyzed Pomace Extracts

Phenol compounds were extracted from whole and skin pomaces. Whole grape pomace and skins separated from dried grape pomace were freezed in liquid nitrogen and grinded with a blender until a fine powder was obtained. In order to evaluate the antibacterial activity of pomaces, whole and skin samples (1 g) were extracted in 10 mL of ethanol:water (40:60, *v:v*) at room temperature for 16 h in the dark under continuous stirring. Extraction mixtures were centrifuged (4000× *g*) for 5 min and the supernatants were dealchoolized by Rotavapor (Buchi, Rotavapor R205, Switzerland) and freeze-dried by a Freezone^®^ 2.5 model 76530 lyophilizer (Labconco Corp., Kansas City, MO, USA) and stored at −20 °C until biological activity analysis. The extracts of whole and skin pomace (WP and SP respectively), of grape cultivars Negramaro (N) and Fiano (F) were lyophilized and suspended in phosphate-buffered saline (PBS, 16.9 mM K_2_HPO_4_, 33.1 mM KH_2_PO_4_) and then analysed for their antioxidant activity (TEAC and ORAC) and the total phenol content (TP). The powder obtained was weighed and suspended in PBS, at a concentration of 500 µg/mL total polyphenols (Gallic acid equivalents). This stock solution was used for antibacterial assays.

### 4.4. High Performance Liquid Chromatography (HPLC) Characterization of Phenols

The phenolic fractions from skin pomaces in hydro-alcoholic extracts and lyophilized samples resuspended in PBS, were separated and quantified through liquid chromatography. RP-HPLC analysis was performed using an Agilent-1100 liquid chromatograph (Agilent Technologies, Italy) equipped with a DAD detector, the separation was performed on C18 column (5 UltraSphere rum spherical 80 A pore, 25 mm), as described by Gerardi et al. [10]. Chromatograms were acquired at 520, 280, 320, 370 and 306 nm. The chromatographic analysis was based on the comparison of peak retention time with the retention time and UV vis spectra of external standards.

### 4.5. Trolox Equivalent Antioxidant Capacity (TEAC) Assay

The TEAC assay of whole and skin pomace samples was performed trough the method reported by Gerardi et al. [8]. The ABTS radical, diluted in PBS (pH 7.4), showed an absorbance value of 0.4 (read at 734 nm). A volume of 200 μL of diluted ABTS was added to 10 μL of extract. Then, the absorbance value was recorder at 734 nm after 6 min using a plate reader (Infinite 200 Pro, Tecan, Männedorf, Switzerland). TEAC values were obtained considering the percentage inhibition at 734 nm with Trolox as standard. TEAC values were expressed as Trolox equivalents (μmol/g) using Magellan v7.2 software (Tecan, Männedorf, Switzerland )).

### 4.6. Oxygen Radical Absorbance Capacity (ORAC) Assay

The ORAC procedure was carried out as per Gerardi et al. [10]. The reaction was carried out using a 96-well plate Infifinite200Pro plate reader (Tecan, Männedorf, Swizerland) in a 75 mM phosphate buffer (pH 7.4), and the final reaction volume was 200 μL. Extracts from dried grape pomace (20 μL) and fluorescein (120 μL; 70 nM, final concentration) solutions were placed in the well of the microplate. The mixture was heated at 37 °C for 15 min. Then, 2,2′-Azobis-(2-methylpropionamidine) dihydrochloride (AAPH) solution (60 μL; 12 mM, final concentration) was added and the fluorescence recorded (excitation and emission wavelengths of 485 and 527 nm, respectively) every minute for 60 min. A blank using phosphate buffer instead of the sample was carried out in each assay and all the reaction mixtures were prepared in triplicate. Decay curves (fluorescence intensity vs. time) were recorded and the net area under the curve was obtained by subtracting the blank value from that of the sample or standard. The degree of antioxidant capacity was quantified using the antioxidant Trolox as a standard (1–6 μM were used to make a standard curve). Final ORAC values were expressed as μmol Trolox equivalents (TE)/g of dried weight of grape pomace.

### 4.7. Folin-Ciocalteu Assay

A rapid method [42] was used to assess the total phenols in alcoholic and water extracts from dried whole and skins pomace, in 96-well plates (Corning) using a microplate reader (Tecan, Infinite M200). Folin-Ciocalteu reagent (1:5, *v/v*) (50 μL) was placed in each well, and then 100 μL of sodium hydroxide solution (0.35 M) was added. The absorbance value at 760 nm was recorder after 5 min of incubation. Gallic acid was used to obtain a calibration curve in the range from 2.5 to 40.0 mg/L (R ≥ 0.9997). Gallic acid equivalents (GAE) were used to express the total phenol content of different samples.

### 4.8. Antibacterial Activity of Grape Pomace Extracts

The antibacterial activity of the whole and skin grape pomace extracts (WP and SP) cv. Fiano (WPF and SPF) and cv. Negramaro (WPN and SPN) was determined against six strains of bacteria purchased from international microbial collections or included in the Agri-Food Toxigenic Fungi Culture Collection (ITEM) of the Institute of Sciences of Food Production (Bari, Italy; http://server.ispa.cnr.it/ITEM/Collection/; accessed on 10 May 2021). The three food spoilage bacterial strains were *Pseudomonas fluorescens* NCPPB 1964^T^, *P. chicorii* ITEM 17296, *P. putida* ITEM 17297 [43,44], whereas the pathogens were *P. aeruginosa* DSM 939, *Staphylococcus aureus* DSM 799, and *Enterococcus faecalis* ATCC 47077. Spoiler strains were cultivated in mPlate Count Broth (mPCB, Becton Dickinson Italia, Milan, Italy) for 24 h at 30 °C, whereas pathogens were cultivated in Brain Heart Infusion (BHI, Biolife Italiana, Milan, Italy) for 24 h at 37 °C. After the incubation, bacterial cell density was adjusted to 0.3 ± 0.05 (*ca.* 10^8^ cfu/mL). Then, cell suspensions were diluted to 10^5^ cfu/mL in mPCB amended with SP or WP at 250 µg/mL total polyphenols. Then, the antibacterial activity of SP extract was further assayed at the final concentration of 250, 125, 62.5, and 31.25 µg/mL total polyphenols. Cell suspensions in mPCB without any grape skin pomace extract were used as controls. Bacterial growth was monitored by measuring optical density every 10 min with the Varioskan Flash (Thermo Fischer Scientific, St. Louis, MO, USA) spectrofluorimeter at a wavelength of 600 nm up to 24 h at 30 °C. Each antimicrobial assay was performed in triplicate. WP and SP extracts from different cultivars were assayed independently in different plates. The Minimum Inhibitory Concentration (MIC) was defined as the lowest SP concentration inhibiting the bacterial growth during the incubation. MICs are expressed in µg of gallic acid equivalents (GAE) per mL of growth medium.

Growth curves were analyzed through the SkanIt™ software (Thermo Fischer Scientific) to calculate the following parameters: maximum growth rate (µmax_ABS_ h^−1^), maximum optical density (ABS_max_), and lag time (λ_ABS_, h).The doubling time (DT, h), defined as the time needed to doubling the OD value starting from the begin of the exponential growth [45], was retrived from growth curves.

At the end of incubation, the cell viability of the strains in the control wells and at selected SPb extract concentrations was assessed through the microdilution plating method [46]. Droplets were seeded on Plate Count Agar (PCA, Biolife Italiana, Milan, Italy) and incubated at 30 °C for 24 h. Viable cell load was expressed as logarithmic unit (log cfu/mL).

### 4.9. Statistical Analysis

The experiments with pomace extracts were conducted in three independent tests, and data were presented as mean ± standard deviation (SD). For chemical analyses, multiple comparisons were carried out by one-way analysis of variance (ANOVA) followed by Tukey’s post hoc comparison tests to establish differences between means (*p* ≤ 0.05). For microbiological tests, growth parameters were compared, for each strain and each SP, through the Tukey test (*p* ≤ 0.05) with the SPSS software (SPSS, Inc., Chicago, IL, USA).

## 5. Conclusions

The comparison of antioxidant and antimicrobial activity of lyophilized whole and skin pomace extracts from red and white grape varieties highlighted differences in antioxidant capacity, phenolic composition, and specific antimicrobial activity against pathogenic and spoilage bacteria. The whole and skin pomace extracts of the Negramaro cv and Fiano cv showed antioxidant and antibacterial activities. Red skin pomace extract of Negramaro cv showed higher antioxidant and antibacterial activity than white skin pomace extract of Fiano cv. Lyophilized grape pomace extracts could be used as natural preservatives in the food industry and as additives in the pharmaceutical/cosmetic sector. Phenols such as flavonoids and soluble phenolic acids can be produced by renewable and low-cost source, exploiting the nutritional and biological value of winery by-products.

## Figures and Tables

**Table 1:** Antioxidant activity (TEAC and ORAC) and Total Polyphenols (TP) of lyophilized extracts of Negramaro (N) and Fiano (F) whole (WP) and skin (SP) grape pomace suspended in PBS.

	WPN	SPN	WPF	SPF
TEAC (μmol TE/g d.w.)	1829.79 ± 137.2 ^a^	651.315 ± 26.91 ^b^	2013.89 ± 65.03 ^a^	479.715 ± 29.52 ^c^
ORAC (μmol TE/g d.w.)	2496.75 ± 449.20 ^a^	1337.75 ± 149.40 ^b^	1767.85 ± 126.96 ^a^	506.273 ± 71.70 ^c^
TP (mg GAEs/g d.w.)	127.87 ± 8.03 ^a^	36.8 ± 5.03 ^b^	127.06 ± 19.01 ^a^	38.01 ± 1.24 ^b^

Mean value ± standard deviation. The same superscript letter in the same row indicate that the mean values are not significantly different (*p* ≤ 0.05).

**Table 2:** Characterization of different classes of compounds occurring in lyophilized extracts of Negramaro (N) and Fiano (F) whole and skin pomace (WP and SP) suspended in PBS and used for antibacterial activity assay.

	WPN	SPN	WPF	SPF
**PHENOL GROUPS**	mg/g Total Phenols			
**Phenolic acids**				
Gallic Acid	1.67 ± 0.30 ^d^	3.58 ± 0.05 ^b^	4.02 ± 0.83 ^a^	1.93 ± 0.22 ^c^
Caffeic Acid	0.72 ± 0.07 ^b^	1.54 ± 0.58 ^a^	n.d.	n.d.
Caftaric Acid	2.17 ± 0.06 ^c^	14.44 ± 0.11 ^a^	2.66 ± 0.32 ^c^	3.39 ± 0.21 ^b^
Coutaric Acid	0.21 ± 0.036 ^c^	0.39 ± 0.006 ^a^	0.31 ± 0.015 ^b^	n.d.
Total	4.77 ± 0.466 ^c^	19.95 ± 0.746 ^a^	6.99 ± 1.165 ^b^	5.32 ± 0.43 ^c^
**Flavanols**				
Catechin	19.2 ± 0.04 ^b^	10.34 ± 1.75 ^c^	26.68 ± 4.84 ^a^	n.d.
Epicatechin	65.1 ± 0.72 ^a^	24.03 ± 4.9 ^b^	50.7 ± 8.93 ^a^	25.95 ± 1.54 ^b^
Total	84.3 ± 0.76 ^a^	34.37 ± 6.65 ^b^	77.38 ± 13.77 ^a^	25.95 ± 1.54 ^c^
**Flavonols**				
Quercetin3-glucoside	1.36 ± 0.09 ^c^	4.93 ± 1.45 ^b^	1.42 ± 0.07 ^c^	16.7 ± 0.57 ^a^
Rutin	2.19 ± 0.21 ^c^	13.04 ± 0.95 ^b^	4.66 ± 0.20 ^c^	64.03 ± 3.47 ^a^
Total	3.55 ± 0.3 ^d^	17.97 ± 2.4 ^b^	6.08 ± 0.27 ^c^	80.73 ± 4.04 ^a^
**Anthocyanins**	mg Malvidin-3-glucoside equivalents/g Total Phenols			
Total	n.d.	107.84 ± 10.3	n.d.	n.d.

Mean value ± standard deviation. For each identified compound, the same superscript letter in the same row indicate that the mean values are not significantly different (*p* ≤ 0.05). n.d.: not detected.

**Table 3:** Mean values (±SD) of the growth parameters of *P. fluorescens* NCPPB 1964^T^, *P. chicorii* ITEM 17296, *P. putida* ITEM 17297, and *P. aeruginosa* DSM 939 grown in mPCB for 24 h at 30 °C with grape pomace skin extract of cv Fiano (SPF, A) or cv Negramaro (SPN, B) (250 µg GAE/mL) and without any supplementation (C, control).

A					
		µmax _ABS_ h^−1^	ABS_max_	λ_ABS_ (h)	DT (h)
*P. fluorescens* NCPPB 1964^T^	C	9.0 × 10^−2^ ± 4.4 × 10^−4 a^	0.76 ± 0.01 ^a^	9.74 ± 0.10 ^a^	2.49 ± 0.04 ^b^
	SPF	1.0 × 10^−2^ ± 3.1 × 10^−4 a^	0.10 ± 0.01 ^b^	9.79 ± 0.05 ^a^	9.75 ± 0.09 ^a^
*P. chicorii* ITEM 17296	C	1.1 × 10^−1^ ± 1.3 × 10^−2 a^	0.90 ± 0.05 ^a^	5.05 ± 0.05 ^b^	1.68 ± 0.05 ^b^
	SPF	3.7 × 10^−2^ ± 2.8 × 10^−3 b^	0.46 ± 0.01 ^b^	5.55 ± 0.14 ^a^	4.93 ± 0.21 ^a^
*P. putida* ITEM 17297	C	6.8 × 10^−2^ ± 3.6 × 10^−4 a^	0.61 ± 0.01 ^a^	6.22 ± 0.10 ^b^	2.10 ± 0.10 ^b^
	SPF	3.3 × 10^−2^ ± 2.7 × 10^−3 b^	0.38 ± 0.03 ^b^	6.95 ± 0.07 ^a^	4.43 ± 0.07 ^a^
*P. aeruginosa* DSM 939	C	7.2 × 10^−2^ ± 2.1 × 10^−3 a^	1.00 ± 0.03 ^a^	8.41 ± 0.07 ^b^	2.14 ± 0.07 ^b^
	SPF	4.9 × 10^−2^ ± 8.5 × 10^−4 b^	0.46 ± 0.01^b^	9.00 ± 0.10 ^a^	3.77 ± 0.10 ^a^
**B**					
		**µmax _ABS_ h^−1^**	**ABS_max_**	**λ_ABS_ (h)**	**DT (h)**
*P. fluorescens* NCPPB 1964^T^	C	6.6 × 10^−2^ ± 3.3 × 10^−4 a^	0.58 ± 0.01 ^a^	9.51 ± 0.07 ^b^	3.26 ± 0.07 ^b^
	SPN *	1.5 × 10^−2^ ± 7.1 × 10^−3 b^	0.14 ± 0.05 ^b^	15.6 ± 1.00 ^a^	5.50 ± 0.05 ^a^
*P. chicorii* ITEM 17296	C	8.8 × 10^−2^ ± 1.7 × 10^−3 a^	0.70 ± 0.01 ^a^	5.49 ± 0.07 ^b^	1.73 ± 0.07 ^b^
	SPN	1.4 × 10^−2^ ± 5.9 × 10^−4 b^	0.15 ± 0.01 ^b^	5.79 ± 0.09 ^a^	6.70 ± 0.09 ^a^
*P. putida* ITEM 17297	C	7.9 × 10^−2^ ± 2.4 × 10^−3 a^	0.62 ± 0.02 ^a^	6.14 ± 0.10 ^b^	1.92 ± 0.10 ^b^
	SPN *	2.6 × 10^−2^ ± 1.6 × 10^−4 b^	0.24 ± 0.01 ^b^	12.5 ± 0.06 ^a^	3.89 ± 0.06 ^a^
*P. aeruginosa* DSM 939	C	9.4 × 10^−2^ ± 1.5 × 10^−3 a^	1.04 ± 0.01 ^a^	8.39 ± 0.07 ^b^	1.60 ± 0.07 ^b^
	SPN	2.5 × 10^−3^ ± 1.2 × 10^−4 b^	0.05 ± 0.01 ^b^	20.5 ± 0.07 ^a^	3.51 ± 0.07 ^a^

* 125 µg GAE/mL. Tukey test was used to compare mean values. Different superscript letters indicate significant differences between C and SP samples (*p* ≤ 0.05).

## Data Availability

Data is contained within the article or Supplementary Materials.

## Supplementary Materials

The following are available online. Figure S1. Growth curves (three replicates) of *P. chicorii*; Figure S2: Anthocyanin profiles in Negramaro skin pomace extract suspendend in PBS analyzed using HPLC; Table S1. The limit of detection (LOD) and limit of quantification (LOQ).

## Sample Availability

Samples of the grape pomace powder are available from the authors.
