# Supplementary material for: Comparison of Antibacterial and Antioxidant Properties of Red (cv. Negramaro) and White (cv. Fiano) Skin Pomace Extracts
Source: Molecules. 2021 Sep 29;26(19):5918. doi: 10.3390/molecules26195918 (PMC8512049; doi:10.3390/molecules26195918)
Supplement: Supplementary file 1 [file molecules-26-05918-s001.zip › molecules-1340090-supplementary.pdf]

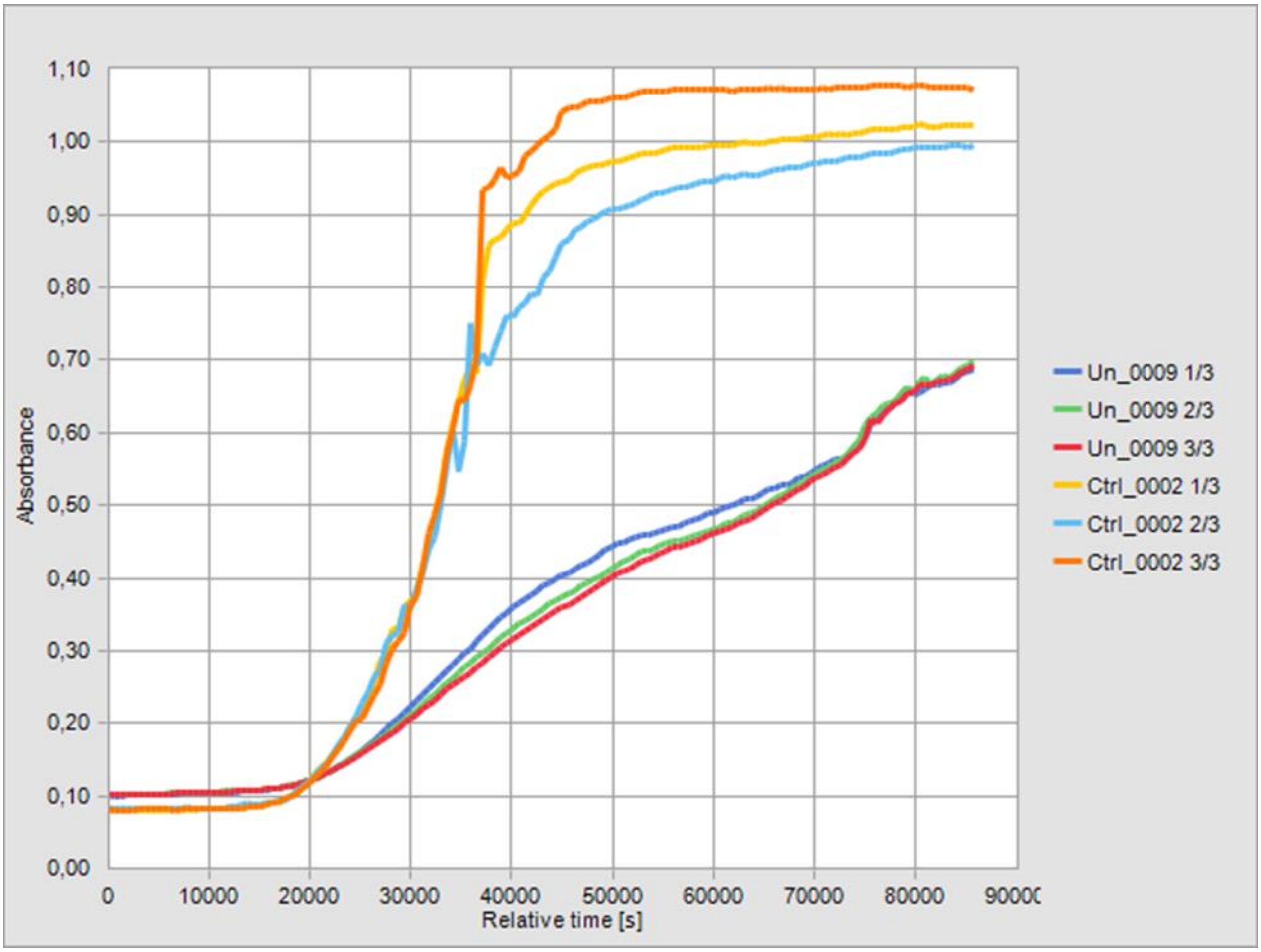

Figure S1. Growth curves (three replicates) of *P. chicorii* ITEM 17296 in mPCB (yellow, light-blue, and orange lines), or mPCB amended with skin pomace extract of cv Fiano (SPF, 250 µg GAE/mL) (dark blue, green, and red lines) during 24 h at 30°C.

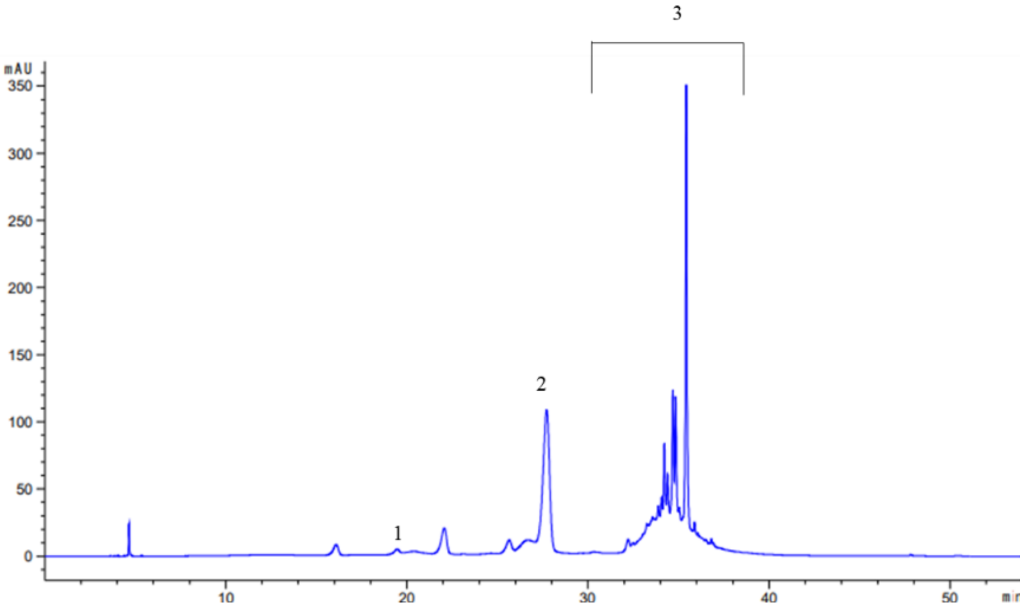

Figure S2. Anthocyanin profiles in Negramaro skin pomace extract suspendend in PBS analyzed using HPLC (λ = 520 nm). Peak 1: 3 cyanidin 3-O-glucoside; Peak 2: malvidin 3-O-glucoside: Peak 3: acylated anthocyanins.

Table S1. The limit of detection (LOD) and limit of quantification (LOQ) were estimated by measuring signal-to-noise ratio (S/N) of the individual peak of each standard compound. The LOD and LOQ were determined as the lowest concentration injected giving S/N ≥ 3 and 10 respectively. Results were expressed as detectable or quantifiable concentrations in micrograms per millilitre (μg/mL).

**Reference Compounds**	**Regression Equation**	**R^2**	**Linear Range (μg/mL)**	**LOD (μg/mL)**	**LOQ (μg/mL)**
Gallic acid	y=76041x-11.964	0.9997	1.0-100	0.26	0.77
Caffeic acid	y=75564x+47.461	0.9987	0.25-100	0.05	0.10
Caftaric acid	y=79756x-16.678	0.9996	1.0-100	0.15	0.55
Coutaric acid	y=112763x+18.373	1.0	1.0-100	0.09	0.23
Catechin	y=17625x+13.792	1.0	1.25-500	0.40	1.02
Epicatechin	y=24972x-15.31	0.9999	1.25-500	0.30	0.89
Quercetin3-gluc	y=40314x-3.718	1.0	1.0-100	0.20	0.59
Rutin	y=31912x+5.256	0.9998	1.0-100	0.20	0.62
Oenin	Y=57005x+16.802	0.9999	1.95-1000	0.23	0.70
